# Multiview Monitoring of Individual Cattle Behavior Based on Action Recognition in Closed Barns Using Deep Learning

**DOI:** 10.3390/ani13122020

**Published:** 2023-06-17

**Authors:** Alvaro Fuentes, Shujie Han, Muhammad Fahad Nasir, Jongbin Park, Sook Yoon, Dong Sun Park

**Affiliations:** 1 Department of Electronics Engineering, Jeonbuk National University, Jeonju 54896, Republic of Korea; afuentes@jbnu.ac.kr (A.F.);; 2 Core Research Institute of Intelligent Robots, Jeonbuk National University, Jeonju 54896, Republic of Korea; 3 Department of Computer Engineering, Mokpo National University, Muan 58554, Republic of Korea

**Keywords:** deep learning, cattle behavior, video, indoor farm, animal welfare, precision livestock farming

## Abstract

**Simple Summary:**

Over the years, the monitoring of cattle behavior has been recognized as an essential aspect of ensuring their health and welfare. In this study, we propose a framework based on artificial intelligence for monitoring the behavior of individual cattle through action recognition and tracking over time. Our research focuses specifically on studying the behavior of Hanwoo cattle, a native breed of Korea. To achieve this, we deployed a network of CCTV (closed-circuit television) cameras strategically placed within a closed farm, showcasing the effectiveness of non-intrusive sensors in capturing real-world information. Furthermore, we devised techniques to tackle challenges such as occlusion, size variations, and motion deformation. Our proposed technology represents a significant advancement in the field of precision livestock farming. By enabling the monitoring of individual animal behavior over time, it offers valuable insights that optimize the management of farms. This innovative approach enhances the efficiency and effectiveness of farm operations, ultimately contributing to the overall success and progress of the agriculture industry.

**Abstract:**

Cattle behavior recognition is essential for monitoring their health and welfare. Existing techniques for behavior recognition in closed barns typically rely on direct observation to detect changes using wearable devices or surveillance cameras. While promising progress has been made in this field, monitoring individual cattle, especially those with similar visual characteristics, remains challenging due to numerous factors such as occlusion, scale variations, and pose changes. Accurate and consistent individual identification over time is therefore essential to overcome these challenges. To address this issue, this paper introduces an approach for multiview monitoring of individual cattle behavior based on action recognition using video data. The proposed system takes an image sequence as input and utilizes a detector to identify hierarchical actions categorized as part and individual actions. These regions of interest are then inputted into a tracking and identification mechanism, enabling the system to continuously track each individual in the scene and assign them a unique identification number. By implementing this approach, cattle behavior is continuously monitored, and statistical analysis is conducted to assess changes in behavior in the time domain. The effectiveness of the proposed framework is demonstrated through quantitative and qualitative experimental results obtained from our Hanwoo cattle video database. Overall, this study tackles the challenges encountered in real farm indoor scenarios, capturing spatiotemporal information and enabling automatic recognition of cattle behavior for precision livestock farming.

## 1. Introduction

Precision livestock farming (PLF) technologies have emerged as innovative concepts aimed at continuously and automatically monitoring animal health and welfare parameters. These technologies offer the potential to enhance productivity and detect health issues at an early stage [1]. Current PLF technologies encompass a range of advancements, including the Internet of Things (IoT), robotics, drones, and artificial intelligence (AI) [2]. In recent years, the study of animal behavior has gained significant attention within the realm of PLF. Researchers have developed more animal-centered approaches that provide insights into how animals behave and interact in their natural environment. These insights serve as valuable indicators for assessing the health, emotions, and overall well-being of animals [3]. Specifically, they can assist in identifying and treating sick animals, responding promptly to immediate issues, selecting animals for breeding, designing appropriate facilities, and managing herds effectively [4]. Consequently, understanding how livestock animals perceive and interact with their environment has become a key concern for stakeholders involved in this domain [5]. In this study, we specifically focus on monitoring cattle behavior for indoor farming purposes.

In livestock tracking, animals are considered complex active systems mainly due to variability in actions and poses, as well as different sizes and sudden movements. Traditionally, monitoring animals in indoor barns has been possible through direct observation [6] or by using wireless wearable devices [7], such as neck collars, ear tags, and leg tags. An extensive literature has demonstrated the utility of these devices [8]. They are primarily equipped with sensors that measure the temperature and activity based on motion obtained from accelerometers [9]. These data provide valuable information for animal behavior [10], health status [11], disease [12], stress [13], and estrus detection [14]. Radio frequency identification (RFID) tags are also used to obtain individuals’ identities. Signals received from these sensors are processed and used by, for instance, classifiers to find the corresponding targets [15]. 

While wearable devices are practical solutions [16], their application often faces several challenges. For instance, data collection strongly relies on communication and battery-powered devices, which are prone to damage, or the devices themselves can cause discomfort to the animals. Moreover, they provide limited information related to only a few specific actions [17]. By comparison, camera-based systems offer promising possibilities for non-intrusive monitoring of animals [18]. Recent works have presented solutions that utilize images or video data obtained directly from surveillance cameras to identify animal actions [19,20,21,22]. In our earlier work [20], we introduced a framework for cattle behavior recognition using video with spatiotemporal information. This framework successfully recognized 15 actions divided into hierarchical groups such as individual, part, and group actions. Individual activities included walking, standing, resting, eating, sleeping, standing up, lying down, and self-grooming. Group activities, such as fighting, feeding, social licking, and mounting, involved more than one individual. Part actions, executed at the individual level, included moving the head, ruminating, and tail wagging. We analyzed cattle herds based on their actions and obtained statistics for specific periods of time, including day and night scenes. 

**Figure 1:**
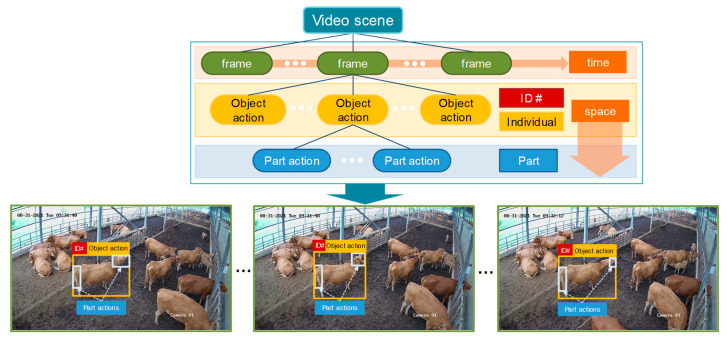
Identification of cattle behavior in the space–time domain. Each individual is identified by an ID number and corresponding actions over time.

Expanding the application of camera-based systems for individual monitoring presents its own set of challenges. These challenges primarily involve issues related to scale deformation, motion variation, and occlusion within the camera’s visible area. Furthermore, obtaining individual identity information over time is necessary to broaden the applicability of vision-based action recognition. In order to overcome these challenges, previous studies explored alternative approaches. One such approach involved evaluating wearable devices and cameras attached to cattle bodies for behavior monitoring [23]. Another study proposed a deep-learning-based approach for cattle identification using video analysis [24]. This approach utilized convolutional neural networks (CNN) and bidirectional long short-term memory (BiLSTM) with a self-attention mechanism, implemented to monitor 50 individuals. The data were obtained from cameras installed on a walking path with visible areas, facilitating their subsequent identification. By comparison, the present study focuses on both action recognition and cattle identification using two CCTV surveillance cameras installed in a closed barn, offering different views and recording times. This represents a more complex and realistic scenario enabling individual monitoring within the actual farm setup where the number of cows remains constant over time. Figure 1 provides examples of camera views targeting cattle in the space–time domain. 

This study presents a strategy for monitoring cattle behavior and identifying individual animals based on deep learning. The data collection setup involved two RGB (red, green, and blue) cameras that covered the closed barn prepared for our study. This setup enabled the collection of video data and the integration of multiple views, allowing for accurate identification and monitoring of a total of 21 cows on the scene. Similar to our earlier work [20], cattle actions are categorized into individual and part actions. However, group actions are only utilized during the detection phase to extract relevant features, but not in subsequent processes, as the primary focus of this research is on monitoring individual animals. The system operates as follows: First, sequential image data is inputted into a deep-learning-based detector, which then outputs the corresponding types of actions and the spatial localization of the cattle. The obtained regions of interest are subsequently fed into a tracker and identification mechanism, which aims to monitor individual animals by assigning them unique identification numbers. Finally, through this implementation, individual statistics and action counts are obtained, allowing for comprehensive monitoring of cattle behavior during specific time periods.

To summarize, the main contributions of the present work are as follows:

- We introduce a deep-learning-based framework for monitoring individual cattle behavior in indoor farms using video data. The complete system not only provides information on the actions performed by the cattle but also enables the identification and tracking of individual animals. We employ an action refinement mechanism with majority voting to post-process the detections, and by controlling the tracking parameters, we achieved optimal results.
- We propose a strategy for data collection and annotation to label each cattle with individual and part actions in the space–time domain. Additionally, we provide specifications for the camera settings, which can be utilized for future implementation and replication of our study.
- We apply a monitoring mechanism to build a database of IDs and actions, allowing for the identification of changes in animal behavior over time through statistical analysis. This objective evaluation enables the detection of normal and abnormal situations by analyzing the actions of each individual.
- The experimental results demonstrate the effectiveness of our proposed approach in our Hanwoo cattle video database, which includes various scenes and recordings captured during different times of the day and night.
- Our research effectively addresses the challenges encountered in real farm scenarios by capturing spatiotemporal information, contributing to the advancement of precision livestock farming.

The remainder of this paper is organized as follows: In Section 2, we introduce our dataset and annotation strategy and provide detailed insights into the proposed framework. Section 3 presents the implementation details and experimental results, showcasing the performance of our model and highlighting the significance of our findings. Finally, in Section 4, we conclude the paper by summarizing the key contributions and suggesting avenues for future research.

## 2. Materials and Methods

### 2.1. Dataset

This study solely utilized video data for monitoring animal behavior. No physical experiments or intrusive devices that could disrupt the animals’ normal conditions were employed in our research. Our primary objective was to develop a technology that enabled us to comprehend animal behavior within their natural environment, thereby enhancing their well-being.

The dataset comprises video recordings obtained from the "Baksagol" private Hanwoo cattle farm located in Imsil, South Korea. This farm is situated in a temperate climate with distinct seasons, experiencing cold, dry winters and hot, humid summers in South Korea. Spring and autumn are relatively brief, offering mild and generally pleasant temperatures. The animal housing facility is designed with semi-open compartments, allowing for external air ventilation. Additionally, each compartment is equipped with indoor ventilators. The floor is covered with sawdust on a basic concrete foundation, while the ceiling consists of opaque Styrofoam steel sheets in some areas and transparent polycarbonate in others. For animal feeding, an automatic feeder dispenses precise amounts based on the diet plan. Furthermore, a robot is employed to automatically distribute forage three times a day—morning, noon, and evening. 

The experimental barn had a size of 30 × 12 meters and housed 21 cows, ranging in age from 1 to 7 years, including 3 calves. To capture continuous video data, two HIK (Hikvision) surveillance camera devices with 4K resolution (3840 × 2160) were installed in the barn. One camera was positioned in a corner of the barn (side angle) to capture a wider view of the surroundings, while the other camera was placed in a central location (north-facing angle) to enable closer observation of the individuals.

**Figure 2:**
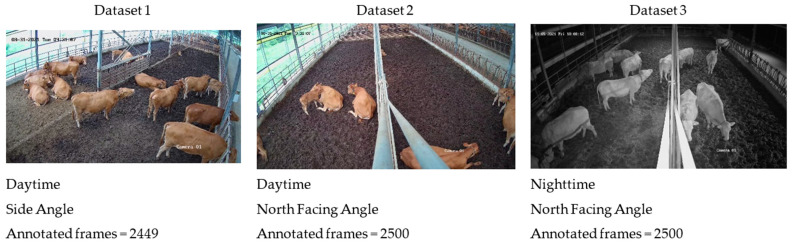
Example images and counts of each dataset from two camera devices capturing day- and nighttime environments at the farm.

We utilized recordings from two camera angles, capturing both day- and nighttime footage, showcasing multiple individuals and their various actions. The baseline model of the detector was constructed using a database comprising a total of 3 videos. To capture more precise variations, images were extracted at a rate of 15 frames per second. Figure 2 provides example images depicting each farm environment. For annotation purposes, a strategy was devised to track individuals and their actions over time, as illustrated in Figure 1. In the space–time domain, cattle were manually identified and assigned unique identification numbers, enabling the recognition of their corresponding actions. Each cow was annotated using a bounding box to indicate its location within the image, the activity performed at that moment, and its assigned identification number. We employed an available online toolbox for this annotation process. The criteria for identifying actions are described in Table 1.

The datasets used in this study consisted of 7449 annotated frames captured from two different angles and during various day and night periods in the cattle barn. Table 2 provides an overview of the number of annotated individuals and activities in each dataset. The annotation process followed the criteria established by [20], where bounding boxes were used to annotate cattle actions within the regions of interest. Cattle actions were categorized into individual and part actions. However, group actions were only utilized during the detection phase to extract relevant features, but not in subsequent processes, as the primary focus of this research is on monitoring individual animals. Furthermore, we employed additional videos from different day and night periods to evaluate the model’s performance in obtaining individual statistics.

### 2.2. Challenges

During the process of data collection and annotation, we encountered several challenges mainly related to pose changes, scale variation, and occlusion. Due to their large bodies and collective behaviors, cattle can be heavily occluded by other cattle. It was often observed that occluded individuals continued to move while in the occluded state and reappeared in different positions. Depending on the distance, angle with respect to the camera, and their postures, they could appear in various areas on the image plane. 

**Figure 3:**
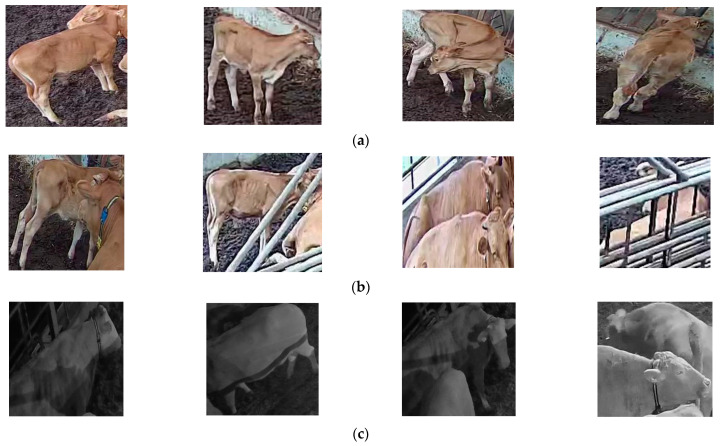
Examples of challenges faced in cattle action recognition within closed indoor environments: (**a**) pose changes; (**b**) occlusion; (**c**) scale and illumination.

Additionally, individuals sometimes remained in crowded groups making their identification difficult. When moving, their actions could change suddenly over time, leading to variations in their visual appearance. Moreover, depending on the time of day, individuals tended to stay in specific areas of the barn, resulting in frequent scale problems when located far from the camera. Lighting variations throughout the day were also a concern. Figure 3 illustrates some examples of the challenges addressed in this study.

### 2.3. General Overview

**Figure 4:**
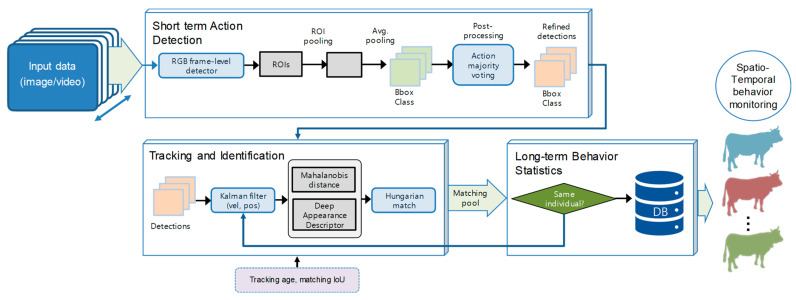
ROI—regions of interest; IoU—intersection over union; vel—velocity; pos—position; DB—Database. Architecture for spatiotemporal action detection and tracking. The detector processes these data and outputs regions of interest corresponding to different actions. These regions of interest are then utilized to initialize the tracker and perform association for cattle identification. The detected actions and assigned identification numbers are accumulated in a database, enabling the derivation of statistics that facilitate the identification of behavior changes over time.

In this paper, we present a framework for cattle behavior monitoring based on action recognition in both short- and long-term spatiotemporal domains. The proposed system operates as follows: First, it takes image data as input to a detector which returns the corresponding actions along with the spatial localization of cattle. To refine the actions, we employ an action refinement mechanism that utilizes majority voting for post-processing the detections and selecting targets accordingly. Subsequently, the obtained regions are used as input to the tracker and identification module, with the objective of monitoring individual cattle in the scene through assigned identification numbers. A matching mechanism is applied to compare previous tracks with new detections for improved accuracy. Finally, through this implementation, we obtain statistics and action counts that facilitate the identification of normal and abnormal cattle behavior during specific time periods in the spatiotemporal domain. Figure 4 shows a representation of the overall context of our research. Each component of the model is described in detail below.

### 2.4. Short-Term Action Detection

In cattle action detection, higher performance is essential for accurately estimating individual statistics through tracking and identification. Therefore, the action detector plays a key role in our approach. Addressing the challenges of missing targets or incorrect detections is of utmost importance in this aspect.

#### 2.4.1. Action Detector

Given an image sequence as input, the detector conducts bounding box detection for individual and part actions. Following our previous approach [20], which uses YOLOv3 (You Look Only Once) as a detector, this paper adopts YOLOv5 [25], along with other techniques to enhance the action recognition performance. YOLO is an end-to-end single-stage object detection algorithm that divides images into a grid array, with each cell in the grid responsible for detecting objects within its region. YOLO has gained significant popularity as an object detection algorithm due to its speed and accuracy, particularly in real-time processing scenarios. YOLOv5 offers several advantages compared to its predecessors, including reduced computational complexity achieved through the use of a cross-stage partial network (CSP), improved performance, especially in detecting smaller objects, owing to its pyramidal-based estimation with a path aggregation network (PANet) and spatial pyramid pooling (SPP) network. These advantages make YOLOv5 highly suitable for our task. 

The detector provides outputs such as the classes of the detected actions, their corresponding bounding boxes, and objectness scores. Our YOLOv5-based model uses binary cross-entropy (BCE) to compute the class and the objectness loss, while complete intersection over union (CIoU) loss is utilized to compute the location loss. Training the detector end-to-end is aimed to minimize the following loss:

(1) $$L=\lambda_{1}L_{cls}+\lambda_{2}L_{obj}+\lambda_{3}L_{loc}$$

where $L_{cls}$, $L_{obj}$, and $L_{loc}$ represent the class, objectness, and localization losses, respectively, and $\lambda_{1}$, $\lambda_{2}$, $\lambda_{3}$ are the weight control parameters.

During the implementation of the action detector, we encountered various challenges primarily associated with the farm environment and lighting conditions. Given the constant background of the barn due to fixed surveillance cameras, it was crucial to address the variability in lighting conditions throughout the day for effective monitoring. To tackle these issues, we applied the following techniques to develop a robust detector capable of detecting cattle actions under diverse visual conditions: 

(1) Data augmentation: Despite having a large dataset, data augmentation played a vital role in object detection by artificially generating new data from existing samples [26]. We employed both soft and strong augmentation techniques to increase the number of labeled samples for action detection and enhance the detector’s robustness against feature variations. Image-based intensity transformations, such as brightness and contrast enhancement, were used to handle illumination changes, while geometric transformations, including horizontal flipping, shearing, and zooming, were applied to address pose variations. Furthermore, strong data augmentation techniques, such as random erasing, random crop, mix-up, cut-mix, and mosaic, were utilized to incorporate partial or occluded features.

(2) Frames per second: To capture the rapid movements of cattle and mitigate the risk of missing targets due to feature variations, we sampled images from the video at a rate of 15 frames per second. These sampled images were used to construct the baseline dataset.

(3) Action majority voting: To enhance action predictions during video inference prior to tracking, we employed action majority voting as a post-processing step. The regions of interest (ROIs) obtained after detection were retained for N−1 consecutive frames to refine the predictions displayed in the Nth frame. By using the bounding box of the first frame as a reference, each box that appeared in all subsequent frames (with an intersection over union, IoU, greater than a specified threshold) had its action determined through voting. This process utilized the class score and the bounding box objectness score, as follows:

(2) $$S_{roi}=\frac{1}{N}\sum_{n=1}^{N}ROI\left({cls}_{n},{obj}_{n}\right)$$

where $S_{roi}$ represents the vote score of the corresponding ROI, and N is the number of frames used for voting and is equal to 4 in this experiment. The action class with the highest number of votes is selected as the final prediction. The computation of the ROI with refined actions can be summarized as follows:

(3) $$R_{roi}={argmax}_{j\in \left\{1,2,3,\ldots ,C\right\}}S_{roi}$$

where $R_{roi}$ denotes the refined ROI with action majority voting and *j* represents the corresponding class in *C*.

#### 2.4.2. Tracking and Identification

**Figure 5:**
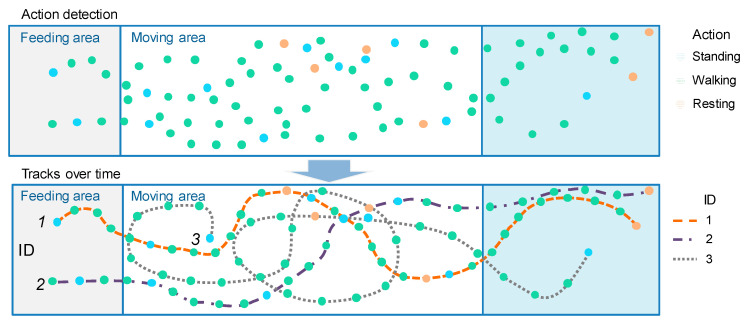
Action recognition and tracking. The detector is responsible for detecting and localizing the actions performed by the animals, while the motion predictor finds the track IDs and establishes associations over time. The final goal of the system is to gather cumulative spatiotemporal statistics that facilitate the identification of changes in animal behavior.

Tracking predicts the positions of cattle throughout the video by leveraging their spatial and temporal features. This is achieved through a two-step approach known as tracking by detection, which consists of action detection for target localization and a motion predictor for future state estimation. The action detector in our system is based on YOLOv5 and has been trained on our cattle activity dataset, as mentioned earlier. The tracker initially receives the set of detections, assigns unique IDs to each detected object, and then tracks them across the video frames while preserving their respective IDs. Once cattle are detected in the first frame, the motion predictor finds their tracks in the spatiotemporal domain. The overall representation of the action recognition and tracking objectives is illustrated in Figure 5.

In the context of cattle tracking, several new challenges arise, particularly in relation to the performance of the detector and its impact on the tracker. For example, when a cow moves towards a crowd and becomes occluded or overlaps with other cows or objects, the tracker should be able to retrieve the same individual when it reappears at a different location. Another challenge is processing speed.

To address these challenges, we explored different multi-object tracking algorithms. Some of the most robust state-of-the-art algorithms in this domain include simple, online, and real-time tracking (SORT) [27], simple online and real-time tracking with a deep association metric (DeepSORT) [28], observation-centric SORT (OCSORT) [29], ByteTrack [30], and StrongSORT [31]. SORT is a pragmatic approach that combines techniques such as the Kalman Filter and the Hungarian algorithm for online and real-time applications. However, SORT may struggle with various poses and occlusions. DeepSORT overcomes these limitations by employing a more robust metric that combines motion and appearance information. 

Figure 3 provides a general overview of the tracker. In our implementation, following DeepSORT, we utilized a standard Kalman filter with constant velocity motion and a linear observation model. First, the bounding box coordinates (u,v,a,h) are used as direct observations of the object state, where (u,v) represent the center of the bounding box, a denotes the aspect ratio, and h indicates the height. The tracker counts the number of frames since the last successful detection and identifies the targets that have left the scene. The initial target association is performed during the first three frames. During this time, the system expects to find correct targets. Tracks that remain unassociated within the next 5000 frames are consequently deleted. To improve prediction accuracy, we controlled the location and velocity parameters of the Kalman filter.

The next step involves associating new detections with the new predictions. For this purpose, we used a Mahalanobis distance to measure the dissimilarity between predicted Kalman states and newly arrived measurements. Thresholding this distance provides the association between detected and predicted tracks. Additionally, to enhance the tracker’s robustness, especially in addressing occlusion issues, we utilized a second metric that computes the Cosine distance between tracks and detections in the appearance space. To obtain the appearance descriptors, we trained a feature extractor offline on our cattle dataset, which provided the feature embeddings used in the cosine metric learning.

(4) $$similarity=\cos\left(\theta \right)=\frac{A\cdot B}{\left\|A\right\|\left\|B\right\|}=\frac{\sum_{i=1}^{n}A_{i}B_{i}}{\sqrt{\sum_{i=1}^{n}A_{i}^{2}}\sqrt{\sum_{i=1}^{n}B_{i}^{2}}}$$

where $A_{i}$ and $B_{i}$ are components of vectors A and B, respectively. 

In combination, both metrics complement each other to achieve efficient tracking results. Mahalanobis distance informs about possible locations based on motion, while Cosine distance utilizes the appearance of the targets. The association between the two distances is determined by a weighted combination, as shown in the following formulation:

(5) $$D=\lambda D_{m}+\left(1-\lambda \right)D_{c}$$

where $D_{m}$ is the Mahalanobis distance and $D_{c}$ is the cosine distance with λ=0.1. 

By incorporating both distance metrics, we establish a robust method for tracking cattle in crowded scenarios. The tracker provides the coordinates of the bounding boxes that indicate the location of the cattle in the image, along with their corresponding detected actions. To further validate the performance of our results, we compared our implementation with the OCSORT, ByteTrack, and StrongSORT trackers, which are other state-of-the-art trackers. 

### 2.5. Long-Term Behavior Analysis

Long-term statistics encompass the information derived from the action detection and cattle tracking processes. The reliability of these systems is crucial for obtaining meaningful data, both in terms of accurately detecting actions and effectively monitoring individual cattle over extended periods. It is of utmost importance to prevent identity switches among individuals, as each action must be correctly associated with its respective individual.

Considering these factors, videos from various time periods were utilized during model inference to assess the system’s ability to handle unexpected movements and occlusion problems in real farm environments. Subsequently, a database was constructed by accumulating the actions attributed to each detected individual.

**Figure 6:**
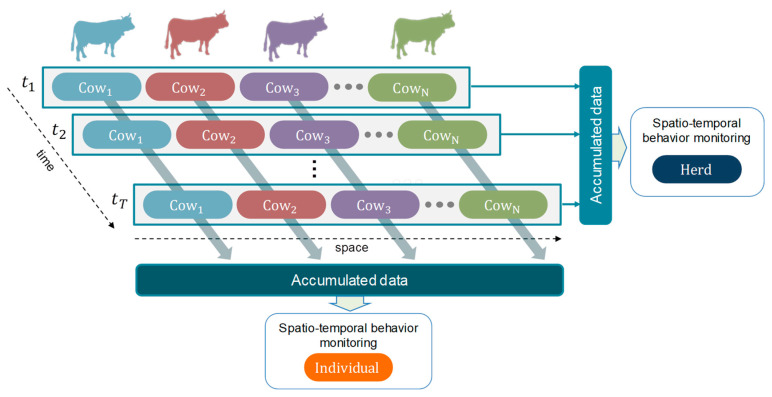
$t_{1}\sim t_{T}$—temporal domain; Spatiotemporal cattle behavior monitoring at individual and herd levels.

Upon completion of the entire inference model, two reports were generated. The first report included the counts of actions associated with each unique ID, providing insights into individual behavior. The second report consisted of trajectories that captured the movement patterns of the cattle throughout the entire evaluation period. Visualization tools were employed to present these dynamics as time series. Figure 6 illustrates the connection between the detection of short-term actions and the identities of individual cattle, which allows for a comprehensive understanding of behavioral changes at both the individual and herd levels using spatiotemporal information. 

## 3. Experimental Results

In this section, we first provide an overview of the implementation details of the system. Subsequently, we present the results of action detection and tracking, which are evaluated using both quantitative and qualitative measures. Finally, we conduct a comprehensive analysis based on the entire process to monitor individuals and herds using spatiotemporal information.

### 3.1. Implementation Details

(1) Action detector: The action detector based on YOLOv5 architecture was trained on our cattle activity dataset using stochastic gradient descent (SGD) on a server PC with four graphic processing units (GPUs) (Titan RTX). The dataset was split into training, test, and validation sets with a ratio of 80:10:10, respectively. Training the model end-to-end aimed to minimize the loss functions presented in Equation (1). Non-maximal suppression (NMS) and intersection over union (IoU) were applied to threshold the area of the final predictions. A confidence threshold of 0.51 and an IoU threshold of 0.5 were used for optimal results. The Adam Optimizer was employed with a scheduled learning rate starting with 0.0001, and a momentum of 0.937. Weight decay and warmup epochs were also incorporated during training.

(2) Tracker: After obtaining the final weights of the action detector, the tracker was implemented using an IoU of 0.75 and a confidence threshold of 0.6 to ensure accurate matching of the predicted target objects. To limit the number of predicted objects per image, a maximum value of 21 was set, which corresponds to the total number of cows in the barn. Additionally, a maximum IoU distance of 0.7 was defined for the predicted boxes within frames. The tracking IDs were assigned an age of 5000 frames, allowing for handling cases where individuals temporarily disappear from the camera view due to occlusion and then reappear later. Cattle traces were compared within a 15-frame window to find the current location while preserving historical location information. If an individual reappeared, the corresponding ID was reassigned; otherwise, a new ID was created. The center of the bounding boxes was used as a reference point for generating a report on cattle trajectories over time. 

(3) Evaluation metrics: The performance of the action detector was evaluated using the mean average precision (mAP). The tracker’s performance was assessed using several commonly used metrics: HOTA (high-order tracking accuracy), MOTA (multi-object tracking accuracy), MOTP (multi-object tracking precision), and IDF1 (identification) [32]. MOTA measures the performance at the detection level between the predicted and ground truth detections in each frame. It also evaluates tracking ID switches and tracks losses. MOTP assesses localization accuracy by averaging the overlap between the correct predictions and the ground truth. IDF1 focuses on association accuracy rather than detection and calculates the mapping between the ground truth and predicted trajectories to determine the presence of unique objects in the scene. HOTA explicitly measures localization, detection, and association, which gives a critical estimation of tracking performance. 

### 3.2. Quantitative Results

#### 3.2.1. Action Detection

The action detection module provides localization information in the form of bounding boxes, along with the corresponding class and objectness information for each cow detected in the scene. To evaluate the performance of our implementation, we compared it with several variants of YOLOv5, ranging from the smallest to the extra-large models. As the model size increases, more parameters are required, and more powerful devices are needed for training. For mobile-based operations, YOLOv5s and YOLOv5m are recommended options due to their efficiency, while for powerful server or cloud computing platforms, YOLOv5l or YOLOv5x are suggested. We conducted experiments using our dataset with all these models and present the results in Table 3. 

In our evaluation, as depicted in Table 3, YOLOv5x achieved the highest mean average precision (mAP) of 95.3%, which is a notable improvement of 2.6% compared to the smallest model. Generally, all trained models demonstrated satisfactory performance in detecting individual actions such as standing, walking, or resting. However, when it came to actions executed at the part level, the models exhibited moderate performance. This can be attributed to the significant variations in these types of actions due to their rapid movement, which occurs locally and on a small scale. 

**Figure 7:**
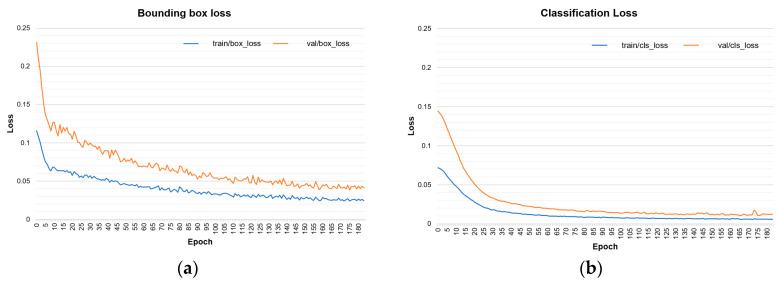

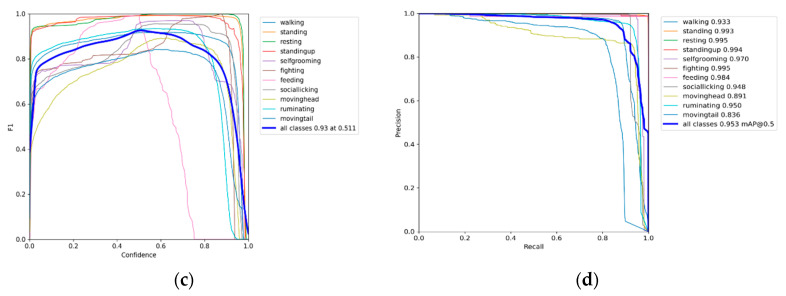
Training curves for action detection: (**a**) bounding box loss; (**b**) classification loss; (**c**) F1- confidence curve; (**d**) precision–recall curve.

Our primary objective was to focus on actions that facilitate the monitoring of animals to identify changes in their behavior. Furthermore, the system possesses the capability to identify group activities involving multiple individuals, including aggressive behaviors such as fighting. In such cases, the system can generate an alert signal to notify the farm manager, enabling them to take immediate corrective actions if necessary. The mAP values are visualized in Figure 7d through the precision–recall curve.

Figure 7 illustrates the learning and evaluation process through the training curves. Overfitting symptoms were mitigated by employing data augmentation techniques, as evidenced in the loss curves for the bounding box and classification. Additionally, the evaluation is presented with the F1 score and the confidence threshold in Figure 7c. It can be observed that most actions were detected with higher precision as the confidence threshold approached 0.51, coinciding with the maximum peak of the F1 score of 93%.

#### 3.2.2. Tracking and Identification

The effectiveness of cattle identification and tracking heavily relies on the quality of the action detector and the corresponding generated weights. Therefore, it is crucial not only to extract the data features but also to properly control the training parameters.

The tracking and identification process focuses on individuals engaged in specific actions over time. To achieve this, we utilized the weights obtained from training the model on the cattle actions database. Specifically, we selected the model with the highest performance to initialize the tracking mechanism. The individuals detected in the first three images of the input video or image sequence were then used to initialize the tracker and enable subsequent monitoring during inference. 

To evaluate the tracking performance, we employed a video containing 12 short video clips, each lasting 3 minutes, captured during day and night periods. In Table 4, we present a comparative analysis of different tracking methods using the aforementioned metrics. This includes a comparison with state-of-the-art trackers such as SORT, DeepSORT, OCSORT, StrongSORT, and ByteTrack, enabling us to select the method that best suits our application. Additionally, we assessed the processing speed by measuring frames per second (FPS).

Based on the findings presented in Table 4, DeepSORT and StrongSORT showed comparable performance with respect to the other tracking methods. However, as mentioned earlier, the action detector via the feature extractor played a crucial role in the predictions. The utilization of ROI action majority voting significantly contributed to the quality of action recognition and bounding box prediction. This can be observed in DeepSORTCattle, where the tracking mechanism exhibited improved stability. 

Managing the age of the tracker was also important, particularly in handling occlusion issues and instances where individuals temporarily disappeared from the scene. Considering the evaluation scenarios’ complexity, controlling the tracker’s age proved beneficial. In terms of metrics, DeepSORTCattle achieved an IDF1 score of 86.05%, representing a comparative advantage over other metrics. Moreover, a total of 53 IDs were obtained after the complete prediction, indicating a noteworthy improvement in long-term tracking. The achieved processing speed was 15 frames per second (fps).

To further support the aforementioned points, Table 5 presents the values used to optimize the tracker’s performance. It was observed that setting a tracking age of 5000 frames, equivalent to approximately 3 minutes, resulted in optimal results for the historical permanence of each bounding box. Additionally, considering the short movement distance observed between frames, the number of unmatched tracks was defined as 7, leading to efficient short-term predictions as well.

**Figure 8:**
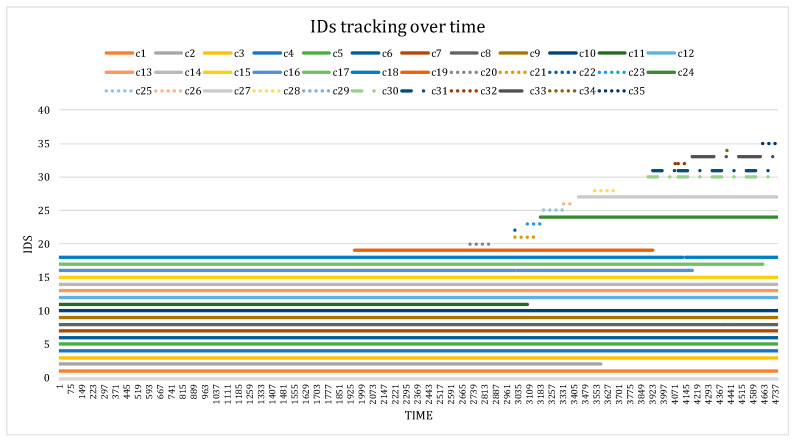
Tracking IDs over time. A total of 21 cows in the barn were detected at different times. ID switches occurred primarily due to occlusion issues. Note: … indicates newly created IDs, and -.-.- represents ID switches.

**Figure 9:**
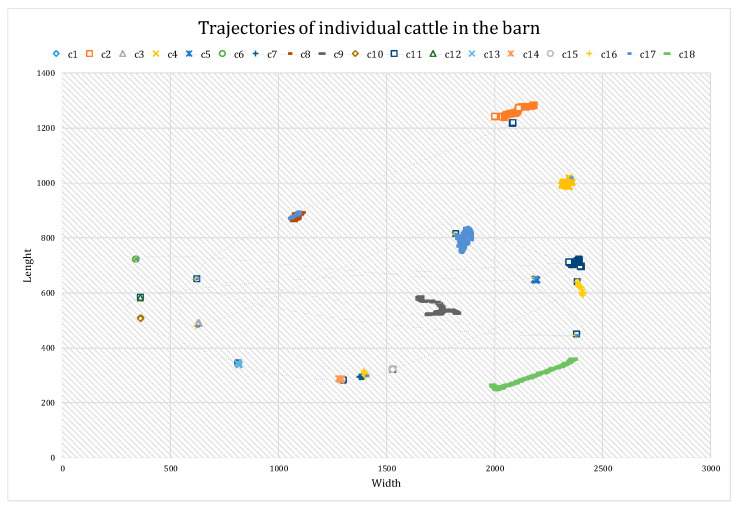
Trajectories of cattle movements in the barn. Cattle movement trajectories within the barn were derived from the center coordinates of the predicted bounding boxes for each cow. These trajectories revealed varied patterns, with some cattle exhibiting active motion through walking, while others remained stationary.

(1) Tracking IDs over time: In addition to the evaluation, we utilized the generated report of IDs to demonstrate the continuity of tracking over time. Using the best DeepSORTCattle model, a total of 53 IDs were obtained, indicating a reduced number of ID switches compared to other trackers. As previously mentioned, the dataset comprised 21 cows, including 3 calves. Figure 8 presents the predictions of the top 35 IDs among the detected ones. We observed consistent tracking performance over time, with minimal ID switches occurring, primarily for the calves. For instance, c16 frequently encountered occlusion issues with other individuals due to its smaller size.

(2) Trajectories in the barn: Another report generated from the results, containing spatiotemporal information, provided the trajectories of the cattle within the barn over a specific time period. Similar to the diagram in Figure 5, these trajectories offer insights into various aspects, such as estimating the distances covered by individuals, enabling the analysis of abnormal behavioral patterns. Utilizing the trajectories derived from the center coordinates of the detected bounding boxes in the sample video, Figure 9 was constructed to depict the trajectories of 18 cows within the barn. While some cows remained stationary, others, such as cows 18 and 11, exhibited more active movements during the evaluation period. It is important to note that this representation serves as an example, utilizing solely image data, and can be extended for continuous monitoring over longer durations.

### 3.3. Qualitative Results

**Figure 10:**
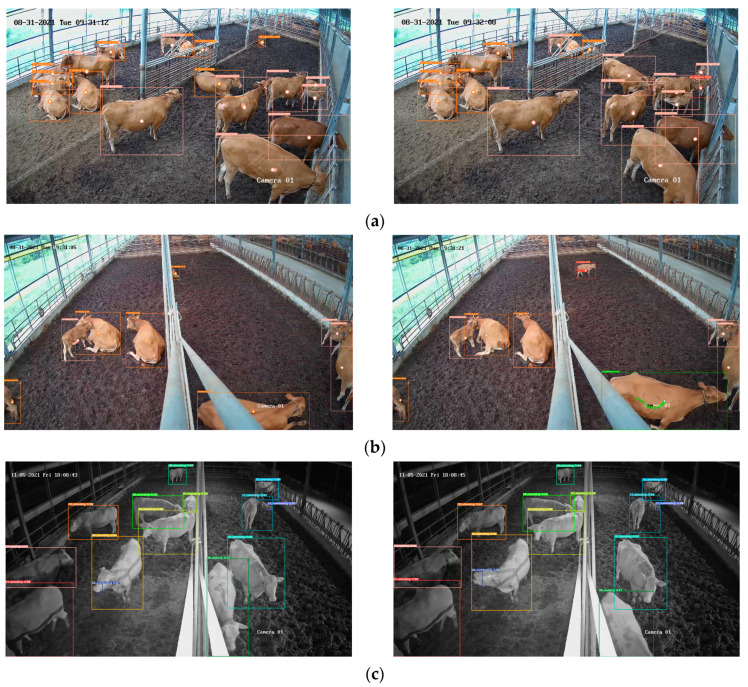
Examples of action recognition over time. Each bounding box shows both the action and the tracking ID. (**a**) Morning scene from camera 1; (**b**) morning scene from camera 2; (**c**) night scene from camera 2. The white point on each bounding box indicates its center. Additionally, the traces for the past 15 frames are displayed, representing historical information.

We applied our proposed framework to various scenes in the test dataset, including both day and night times, to visualize the predicted targets in the images. The action detection algorithm successfully detected actions and assigned IDs to each individual. Figure 10 showcases some examples of the qualitative results, where each bounding box displays the detected action and corresponding tracking ID. These examples demonstrate the system’s ability to track individuals and monitor their actions over time. It is worth noting that actions occurring during the day were easier to identify due to favorable lighting conditions, while at night, the cattle mostly remained in a resting state. Thus, our system demonstrates a capability to generalize well across different environments, achieved through training the detector using images from various cameras and environments and leveraging the robust tracking mechanism to maintain targets throughout the evaluation period.

To further evaluate the performance of the proposed framework, we utilized tSNE (t-distributed stochastic neighbor embedding) distributions generated from the regions of interest obtained during the detection process. These distributions serve as indicators of the system’s effectiveness in detecting individual actions while also highlighting the challenges associated with part detection. 

**Figure 11:**
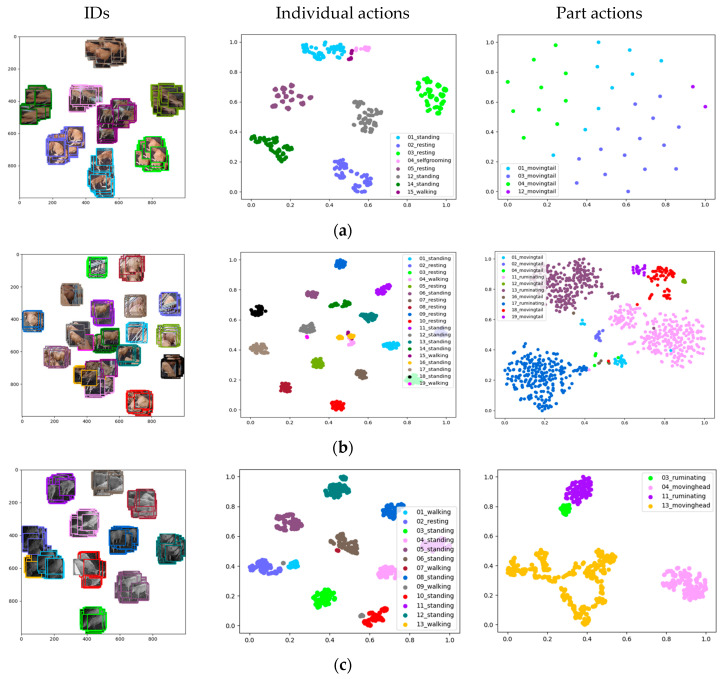
tSNE distributions of the regions of interest (ROIs) obtained from action detection and IDs evaluated during day- and nighttime. The distributions are presented from left to right, showcasing the IDs, individual actions, and part actions. (**a**) Morning scene from camera 1; (**b**) morning scene from camera 2; (**c**) night scene from camera 2.

The tSNE distributions revealed that the system performed well in distinguishing individual actions, as evidenced by the presence of compact regions and accurate matches distributed across the space. However, it encountered challenges in the detection of part actions, resulting in closer distributions. This difficulty primarily arose from identifying specific part actions such as tail wagging or smaller regions associated with rumination, which often appeared similar across different cows. Figure 11 showcases selected scenes and their corresponding tSNE distributions, offering a visual analysis of the system’s performance in action detection and the challenges it faced in part detection. 

### 3.4. Long-term Behavior Analysis with Spatiotemporal Data

By analyzing the accumulated actions performed by each individual, it was possible to derive statistics that reveal behavior patterns over specific time periods. The implemented system enabled the extraction of such statistics based on the detections obtained. The analysis below illustrates the potential for obtaining statistics at both the herd level and for individual animals. In this example, a four-minute video captured by one of the farm’s cameras, representing a scene from 9:01 to 9:05 am, was used for analysis.

**Figure 12:**
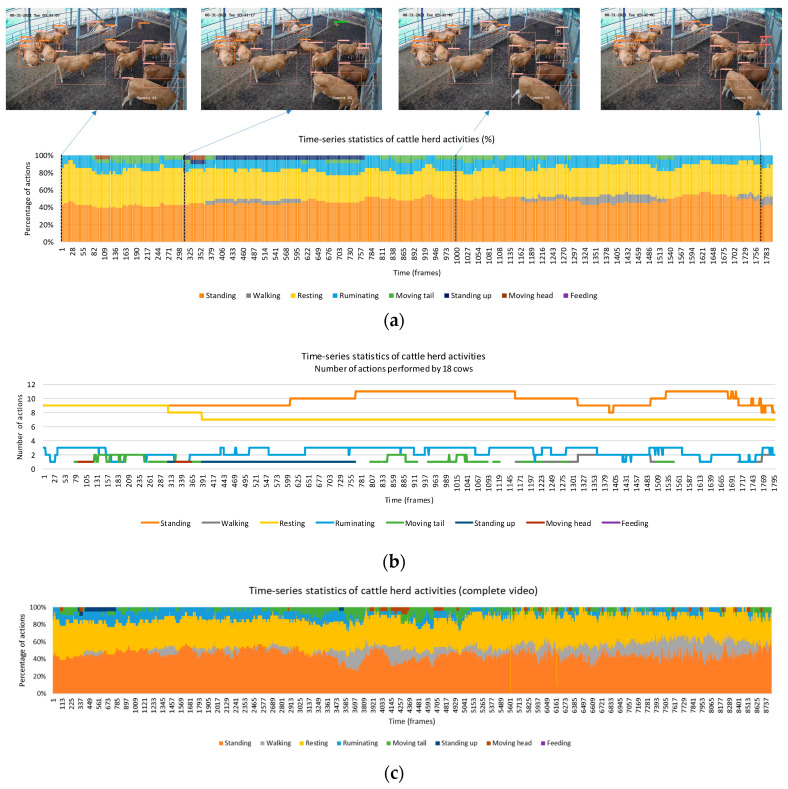
Time-series statistics of the cattle herd shown for a one-minute period, focusing on the percentage of actions (**a**), and the number of actions (**b**). The illustration is extended to cover the entire four-minute video (**c**), providing a comprehensive overview of the herd’s behavior over time. Individual and part actions are included in the graphs.

**Figure 13:**
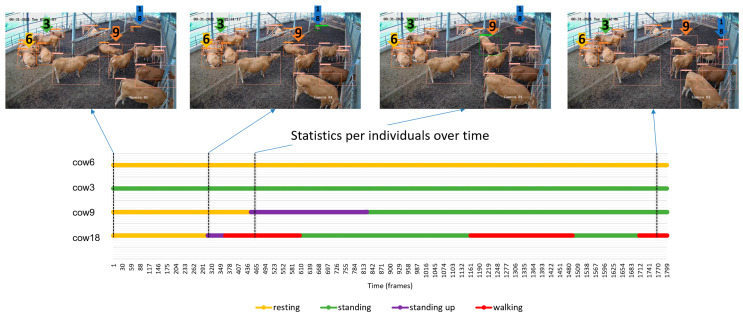
Time-series statistics of individual cattle for a one-minute period. Four cows with both active and quiet behaviors were specifically chosen to demonstrate these variations. The numbers 3, 6, 9, and 18 represent the zoom of the selected cattle IDs assigned by the model in the initial frame.

(1) Herd statistics. Figure 12 depicts the analysis of the overall herd behavior. The actions are presented in two ways: the percentage of occurrence (Figure 12a) and the number of individuals involved (Figure 12b). For instance, during the monitoring period, it was observed that approximately 40% of the total animals were standing, another 40% were resting, around 10% were engaged in rumination, and occasional actions such as standing up, walking, and tail wagging (moving tail) were also observed. To expand the analysis, the entire 4-minute video was considered, as shown in Figure 11c. This longer duration provides a more comprehensive view of the herd’s behavior, with greater emphasis on actions such as standing, resting, and walking. 

(2) Individual statistics. In addition to analyzing the herd as a whole, statistics were generated at the individual level by utilizing the identification numbers assigned by the tracker. This approach relied on the stability of tracking and the accurate detection of individual actions. Figure 13 provides an example of the statistics obtained for four selected cows, chosen for their diverse range of actions. For instance, cow c18 exhibited the most activity, transitioning from resting to standing up, followed by walking, and then returning to a standing position. On the contrary, cow6 remained in a resting state throughout the entire period. The information depicted in this figure showcases how statistics can be derived using image data for real-time monitoring of individual animals over time. However, it is important to note that the effectiveness of the detector and tracking mechanism plays a critical role. Thus, the quality of the database and subsequent processes for action detection and identification number assignment is of utmost importance.

### 3.5. Discussion

Conducting behavior studies on cattle provides producers with accurate insights into individual animals, enabling a comprehensive understanding of their behavior patterns over time. This practical approach greatly enhances farm management by facilitating prompt information gathering and timely responses to identified issues. Consequently, it optimizes resource allocation, improves animal well-being, and reduces losses resulting from factors such as disease or animal stress.

In contrast to previous studies utilizing wearable devices, the present study introduces a non-intrusive technique that leverages video data. This novel approach involves installing multiple cameras on a real farm, capturing data over time, and promoting the use of non-intrusive information for monitoring animal behavior. The collected video data is then processed by a deep-learning-based architecture, which provides insights into animal behavior and individual identification over time. The derived data enables the generation of statistical analysis, offering a comprehensive understanding of animal behavior through their actions.

The qualitative and quantitative results obtained from both action detection and tracking validate the effectiveness of the proposed techniques across various scenarios. Furthermore, employing state-of-the-art trackers and metrics allows for comparative analysis, revealing significant potential for further improvements in future research. However, an important limitation of the current model is the impact of the action detector on the tracker’s performance. Addressing this limitation requires obtaining additional feature variations from the data. Moreover, video data annotation has proven to be a complex and time-consuming task. Hence, additional research and techniques are necessary to replicate the proposed framework as a more generalized system capable of operating across multiple farms. Additionally, the integration of other sensor devices can contribute to the study of animal behavior and the identification of stress factors. Our future research endeavors will aim to tackle these challenges.

## 4. Conclusions

This paper introduced a deep-learning-based approach for automated monitoring of individual cattle behavior. By detecting actions and generating tracking IDs from multiview video data, we obtained valuable spatiotemporal information over time. The proposed method utilized an image sequence as input to a detector, which categorized actions into part and individual actions. These regions of interest were then fed into a tracking and identification mechanism to assign identification numbers to each individual in the scene. Optimal results were achieved through an action majority voting mechanism for regions of interest and careful control of tracker parameters. Through this implementation, cattle behavior was effectively monitored during specific time periods, and statistical analysis enabled the assessment of behavior changes. We demonstrated the effectiveness of our approach through quantitative and qualitative evaluations using the Hanwoo cattle video database, encompassing both day and night recordings. Our study presented a practical strategy for precision livestock farming, employing non-intrusive surveillance cameras. In practice, the study of animal behavior generates benefits for the producer, not only in terms of obtaining highly accurate and individual-oriented behavior for animal well-being but also in facilitating farm management and resource optimization. Furthermore, the approach could be further extended to monitor dairy cows and other livestock animals as well.

## Figures and Tables

**Table 1:** Criteria for cattle activity annotation.

Category	Actions	Criteria for Visual Identification	Target Area
Individual	Standing	Legs are straight, supporting the body	Body
	Walking	Moving in a standing position	Body
	Resting	Lying with their legs folded underneath them	Body
	Standing up	Action from lying down to standing	Body
	Self-grooming	Licking its own body with the tongue	Body
Part	Moving head	Head tilt, downward or sideways	Head
	Ruminating	Chewing, mouth movements	Mouth
	Tail wagging	Tail movements from side to side	Tail
Group	Social licking	Licking another’s body with the tongue	Body
	Fighting	Head butt between two individuals	Body
	Feeding	Cow feeding a calf	Body

**Table 2:** Datasets of cattle individual and part actions.

Actions	Dataset 1	Dataset 2	Dataset 3	Total Counts Per Action
Standing	25,585	10,853	25,044	61,482
Walking	3305	1560	1562	6427
Resting	18,768	7080	456	26,304
Standing up	243	284	-	527
Self-grooming	247	224	-	471
Ruminating	5430	1241	4161	10,832
Tail wagging	3505	1998	13	5519
Moving head	308	231	660	1199
Total	54,086	23,471	31,896	112,761

The values represent the number of annotated bounding boxes.

**Table 3:** Action detection results.

Actions	Category	Object Detectors			
		YOLOv5s	YOLOv5m	YOLOv5l	YOLOv5x
Standing	Individual	99.2	99.3	99.2	99.3
Walking		89.8	91.8	91.6	93.3
Resting		99.5	99.5	99.5	99.5
Standing up		99.5	99.3	99.4	99.4
Self-grooming		77.6	82.1	95.7	97.0
Moving head	Part	89.7	88.9	89.6	89.1
Ruminating		92.2	94.2	94.0	95.0
Tail wagging		81.6	83.2	82.7	83.6
Social licking	Group	92.2	94.0	93.1	94.8
Fighting		99.5	99.5	99.5	99.5
Feeding		99.1	98.3	99.2	98.4
Total mAP		92.7	93.6	94.9	95.3

**Table 4:** Comparison with state-of-the-art object trackers.

Method	HOTA (↑)	IDF1 (↑)	MOTA (↑)	MOTP (↑)	IDs (↓)	FPS (↑)
SORT	55.10	65.12	63.10	70.60	312	75
OCSORT	65.20	76.28	75.30	72.40	112	115
ByteTrack	63.40	78.13	78.90	73.10	87	20
StrongSORT	76.03	86.04	78.61	83.18	76	7
DeepSORT	73.54	84.33	77.35	73.22	120	15
DeepSORTCattle *	78.50	86.05	79.82	81.20	53	15

* Parameter tuning and ROI vote average; **↑**—higher is better; **↓**—lower is better

**Table 5:** Hyperparameter tuning.

DeepSORTCattle	ROI Vote	Age	Unmatched Tracks	HOTA (↑)	IDF1 (↑)	MOTA (↑)	MOTP (↑)	IDs (↓)
DeepSort		100	7	73.54	84.33	77.35	73.22	120
✓*	✓	100	2	70.17	76.52	72.25	71.52	140
✓*	✓	500	7	71.23	78.17	72.49	75.36	110
✓*	✓	1000	7	75.01	83.28	75.13	80.25	79
✓*	✓	5000	7	78.50	86.05	79.82	81.20	53

* DeepSORTCattle; ✓—with ROI vote; **↑**—higher is better; **↓**—lower is better.

## Data Availability

The data presented in this study are available on request from the corresponding author. The data are not publicly available due to project restrictions.
